# Predicting Lapatinib Dose Regimen Using Machine Learning and Deep Learning Techniques Based on a Real-World Study

**DOI:** 10.3389/fonc.2022.893966

**Published:** 2022-06-03

**Authors:** Ze Yu, Xuan Ye, Hongyue Liu, Huan Li, Xin Hao, Jinyuan Zhang, Fang Kou, Zeyuan Wang, Hai Wei, Fei Gao, Qing Zhai

**Affiliations:** ^1^Institute of Interdisciplinary Integrative Medicine Research, Shanghai University of Traditional Chinese Medicine, Shanghai, China; ^2^Department of Pharmacy, Fudan University Shanghai Cancer Center, Shanghai, China; ^3^Department of Oncology, Shanghai Medical College of Fudan University, Shanghai, China; ^4^Dalian Medicinovo Technology Co., Ltd., Dalian, China; ^5^Beijing Medicinovo Technology Co., Ltd., Beijing, China; ^6^Faculty of Engineering, School of Computer Science, The University of Sydney, Sydney, NSW, Australia

**Keywords:** lapatinib, machine learning, deep learning, TabNet, breast cancer, real-world study, individualized medication model

## Abstract

Lapatinib is used for the treatment of metastatic HER2(+) breast cancer. We aim to establish a prediction model for lapatinib dose using machine learning and deep learning techniques based on a real-world study. There were 149 breast cancer patients enrolled from July 2016 to June 2017 at Fudan University Shanghai Cancer Center. The sequential forward selection algorithm based on random forest was applied for variable selection. Twelve machine learning and deep learning algorithms were compared in terms of their predictive abilities (logistic regression, SVM, random forest, Adaboost, XGBoost, GBDT, LightGBM, CatBoost, TabNet, ANN, Super TML, and Wide&Deep). As a result, TabNet was chosen to construct the prediction model with the best performance (accuracy = 0.82 and AUC = 0.83). Afterward, four variables that strongly correlated with lapatinib dose were ranked *via* importance score as follows: treatment protocols, weight, number of chemotherapy treatments, and number of metastases. Finally, the confusion matrix was used to validate the model for a dose regimen of 1,250 mg lapatinib (precision = 81% and recall = 95%), and for a dose regimen of 1,000 mg lapatinib (precision = 87% and recall = 64%). To conclude, we established a deep learning model to predict lapatinib dose based on important influencing variables selected from real-world evidence, to achieve an optimal individualized dose regimen with good predictive performance.

## Highlights

1. What is the current knowledge on the topic?

Lapatinib was approved in China to treat patients with HER2(+) metastatic breast cancer in combination with capecitabine based on a single-arm, open-label study (EGF10949). Two dose regimens are commonly recommended for lapatinib, 1,250 mg of lapatinib in combination with capecitabine and 1,000 mg of lapatinib in combination with trastuzumab. Under the recommended dose regimen, lapatinib can be well tolerated with minimal avoidance of drug toxicities.

2. What question did this study address?

In this study, we established a deep learning model to predict the lapatinib dose based on important influencing variables from real-world evidence, resulting in getting the optimal individualized dose regimen.

3. What does this study add to our knowledge?

This study provides a new perspective and guidance for lapatinib dose administration where few studies focused on individualized lapatinib dose treatment in breast cancer patients previously.

4. How might this change clinical pharmacology or translational science?

Models based on machine learning and deep learning methods could help clinicians treat breast cancer patients with individualized lapatinib dose regimens to get the optimal effect and reduce adverse events.

## Introduction

Lapatinib is a selective inhibitor of the tyrosine kinase receptor and human epidermal growth factor receptor-2 (HER2) and has activity in HER2-overexpressing breast cancer (1–3). By binding to the ATP-binding site of the receptor’s intracellular domain, lapatinib blocks HER2 tyrosine kinase activity leading to inhibition of tumor cell growth (4). After the progress with anthracycline, taxane, and trastuzumab in China, lapatinib has been introduced for the treatment of advanced/metastatic HER2(+) breast cancer (1, 4–6). Two dose regimens are commonly recommended for lapatinib, which can be optimally tolerated (7). For patients with advanced HER2(+) breast cancer progressing with therapy with anthracyclines, taxanes, and trastuzumab, it is recommended to administer 1,250 mg of lapatinib in combination with capecitabine (4). For patients with metastatic HER2(+), hormone receptor(-) breast cancer upon progressing with therapy with trastuzumab and chemotherapy, it is recommended to administer 1,000 mg of lapatinib in combination with trastuzumab (4). Under the recommended dose regimen, lapatinib can be well tolerated with minimal avoidance of drug toxicities, which are skin rash and diarrhea predominantly (6, 8–10). Therefore, a promising model to predict an appropriate individualized dose regimen is important to get a balance of lapatinib efficacy and toxicities to improve the treatment outcome.

With the rapid development of information technology, real-world study has become an important data source for clinical research (11). Most real-world studies use information from electronic medical records, examination data, and follow-up records during diagnosis and treatment. Real-world study is a process of data mining, model building, and clinical feature data extraction. The main advantages of real-world studies include rich evidence resources, good external validity, individualized program application, and being closer to clinical practice (12, 13). Compared with conventional modeling methods, machine learning and deep learning techniques have indubitable advantages in dealing with real-world evidence, such as the following: (1) machine learning and deep learning can deal with more complex, high-dimensional, and interactive variables, which is lacking in traditional models, and (2) machine learning and deep learning models have stronger generalization and better accuracy than traditional models (14–16). Recently, some algorithms with more sophisticated principles have been developed, such as eXtreme Gradient Boosting (XGBoost), light gradient boosting machine (LightGBM), Categorical Boosting (CatBoost), Gradient Boosting Decision Tree (GBDT), and TabNet, which have been highly recognized in algorithm competitions (17–21). Recently, the application of machine learning and deep learning techniques based on real-world study has been a trend, such as a novel prognostic scoring system of intrahepatic cholangiocarcinoma with ensemble machine learning algorithms (XGBoost, random forest, and GBDT), a prediction model of tacrolimus blood concentration in patients with autoimmune diseases using XGBoost, a novel vancomycin dose prediction model through XGBoost, and warfarin maintenance dose prediction through LightGBM (22–25). Many studies have demonstrated the advantages of machine learning algorithms over traditional statistical methods. With the increasing number of input subject data, machine learning and deep learning models can continually optimize parameters to achieve better performance and practicality.

In order to achieve a balance of drug efficacy and toxicities, an appropriate dose regimen is important for patients’ treatment outcome. In this study, we aim to establish a model based on machine learning and deep learning techniques to predict the lapatinib dose based on important influencing variables from real-world evidence, resulting in getting the optimal individualized dose regimen.

## Methods

### Study Population

This is a retrospective, real-world study. Patients who were diagnosed with breast cancer and treated with lapatinib were included from July 2016 and June 2017 at Fudan University Shanghai Cancer Center (FUSCC). One hundred fifty-four patients were enrolled, including 55 at the initial dose of 1,000 mg, 94 at the initial dose of 1,250 mg, and 5 at the initial dose of 500 mg. This study mainly considered patients with commonly recommended dose regimens, namely, 1,000 mg and 1,250 mg. Therefore, after excluding patients with an initial dose of 500 mg, 149 patients remained. This study was approved by the ethics committee (No. 2016-106-1159-K1), and informed consents were included.

### Data Collection and Processing

All data were collected from electronic medical records. Demographic information included continuous variables, such as age, height, and weight, and binary variables, such as age ≥52 years or not. Combination medication information included the prior use of anthracycline, taxane, platinum, fluorouracil, and trastuzumab. Physiopathological conditions indicated that patients had hypertension, diabetes, heart disease, other underlying diseases (including small samples of epilepsy, hepatitis, hyperthyroidism, chronic enteritis, Hashimoto’s thyroiditis, hepatitis B), and postmenopausal or not. Treatment protocol information included number of previous chemotherapy regiments, Ki-67, prior endocrine therapy, estrogen receptors (ER), progesterone receptors (PR), disease stage, operation, Eastern Cooperative Oncology Group (ECOG), number of metastases, lung metastases, liver metastases, bone metastases, brain metastases, protocol_1 (combination regimen of lapatinib + capecitabine), protocol_2 (combination regimen of paclitaxel + carboplatin + herceptin + lapatinib), protocol_3 (combination regimen of vinorelbine + lapatinib), and protocol_4 (other combination regimens).

There were two initial dose regimens of lapatinib, 1,000 and 1,250 mg, which were converted to binary variables, where 1,250 mg corresponds to “1” and 1,000 mg corresponds to “0.” According to the National Cancer Institute Common Terminology Criteria for Adverse Events (NCI-CTCAE, version 5.0), patients with adverse drug reactions of grade ≤ 2 were considered to have drug safety. In addition, according to a previous study on lapatinib in breast cancer patients at FUSCC, the median progression-free survival (PFS) was 8.1 months; therefore, patients with PFS >8.1 months were considered to have drug effectiveness herein (1). The safety and effectiveness of the drug regimen were also converted to binary variables, where patients showing both safety and effectiveness correspond to “1,” and other situations (either showing safety or effectiveness; not showing safety or effectiveness) correspond to “0.” The target variable was the initial dose regimen of lapatinib (1,000 or 1,250 mg). The variables with extremely imbalanced positive and negative sample sizes in the dataset were eliminated. In terms of data with missing values, the variables were interpolated by the random forest algorithm through learning information about similar patients.

### Variable Selection and Model Establishment

**Figure 1:**
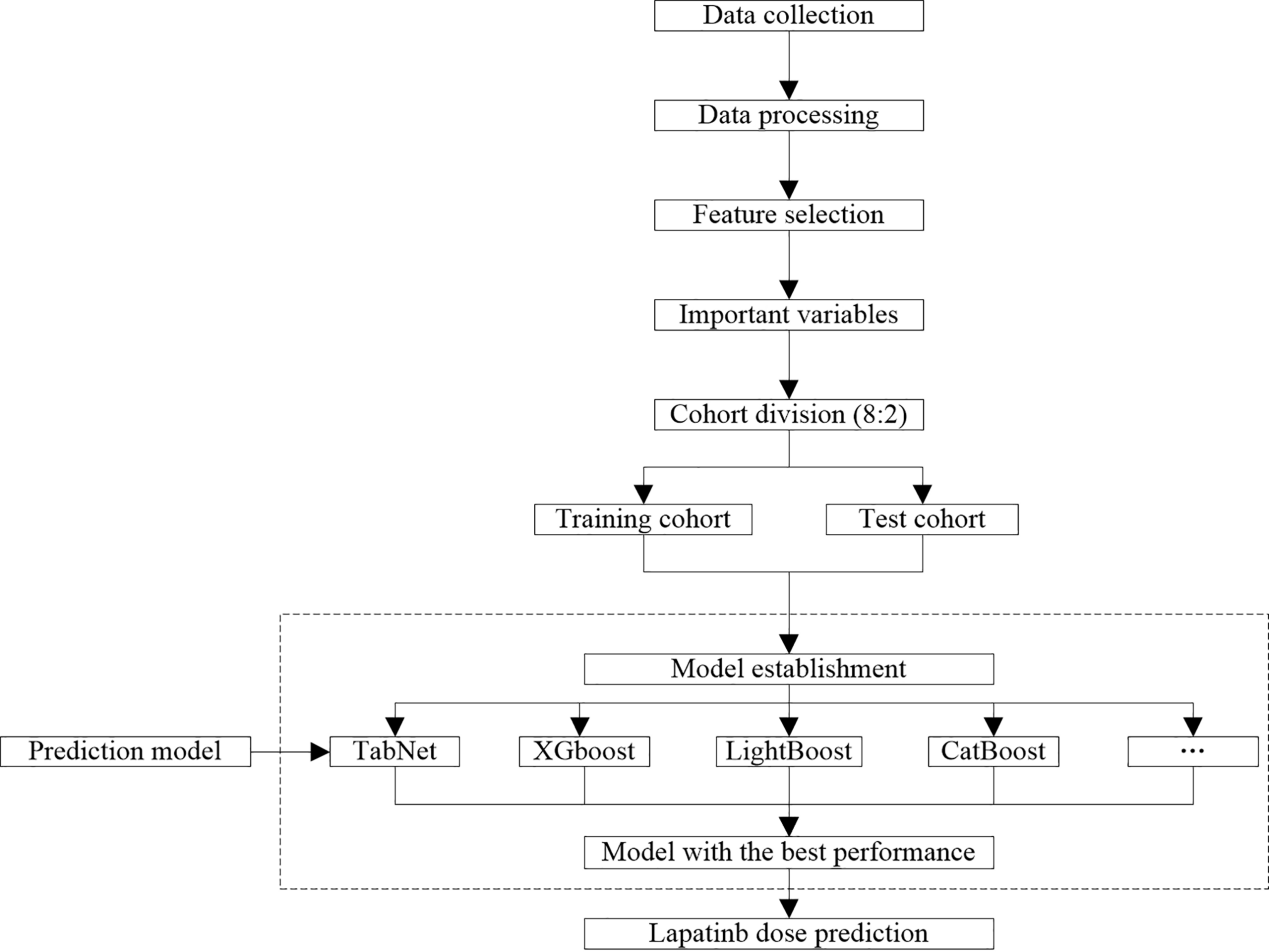
Workflow of data process and model establishment.

The modeling process is illustrated in **Figure 1**. After collecting and processing data of all eligible samples, the sequential forward selection (SFS) algorithm based on RF was applied for selecting the minimum size and optimum performance of the feature subset (26). The SFS algorithm added one feature to the feature subset each time, iteratively generated a new model, and calculated the model performance (f1_score). F1_score is a comprehensive evaluation index of precision and recall, and higher f1_score indicates better model robustness. The iteration stopped when f1_score of the feature subset reached the optimal value. The feature subset with the minimum size and optimum f1_score was therefore selected.

The training cohort and test cohort were divided according to 8:2. The dose prediction model was established and compared by 12 algorithms, which were algorithms with good predictive ability in various common algorithm types, including logistic regression, support vector machine (SVM), random forest (RF), Adaboost, XGBoost, gradient-boosted decision tree, LightGBM, CatBoost, TabNet, Artificial Neural Network (ANN), Super TML, and Wide&Deep, respectively. As a novel deep learning architecture, we implemented the TabNet model exactly as described in Arik and Pfister, used a sparsemax attention, and included the sparsification term in the loss function (21). The model-specific hyperparameters were n_d = 8, n_a = 8, n_steps = 3, gamma = 1.3, cat_emb_dim = 1, n_independent = 2, n_shared = 2, epsilon = 1e-15, momentum = 0.02, lambda_sparse = 0.001, seed = 0, clip_value = 1, verbose = 1, max_epochs = 200, virtual_batch_size = 16, batch_size = 64. TabNet architecture and implementation details are illustrated in **Supplementary Figures S1**–**S4**.

### Statistical Analysis

Subsequently, based on the selected important variables, the evaluation metrics for model performance were calculated, including precision, recall, f1_score, accuracy, and area under the curve (AUC). The model with the best predictive performance in the test cohort was selected to predict the lapatinib dose regimen. The specific formula of evaluation metrics are as follows:

Accuracy = (TP + TN)/(TP+FN+FP+TN)

Precision = TP/(TP + FP)

Recall = TP/(TP + FN)

f1_score = 2 × TP/(2 × TP + FP + FN)

TP: true positive, indicating that the positive class is predicted as the number of positive classes; TN: true negative, indicating that the negative class is predicted as the number of negative classes; FP: false positive, indicating that the negative class is predicted as the number of positive classes; FN: false negative, indicating that the positive class is predicted as the number of negative classes.

f1_score is used to measure the merits and defects of the model; higher f1_score indicates better model performance.

The importance of variables refers to the degree to which each variable in the model contributes to improving the predictive power of the whole model. Herein, we used the algorithm with the best model performance to calculate and rank the variable importance scores. In terms of importance score calculation and ranking by TabNet, the Feature Transformer layer realizes the calculation and processing of features selected by the current step. Analogous to a decision tree, for a given set of features, a decision tree constructs a combination of size relations of individual features, namely, a decision manifold. A simple neural network is used to simulate the decision manifold of the decision tree through a fully connected (FC) layer, but the FC layer constructs a set of simple linear relations and does not consider more complicated cases. TabNet performs feature calculation through a more complex Feature Transformer layer. Its decision manifold may not be similar to that of the decision tree, and it may do better than the decision tree in some Feature combinations (21). Univariate analysis was performed through the Mann–Whitney U test on continuous variables and the chi-square test on classified variables.

Eventually, the confusion matrix was used to visualize the performance of the algorithm and further analyze the model performance. The confusion matrix was realized by the Matplotlib package. All experiments of machine learning and deep learning algorithms were run on Windows 10 with Intel(R) Core(TM) i5-10400F CPU @ 2.90GHz 12CPUs and 512GB memory. Data analysis was conducted using python 3.8.8 and IBM SPSS Statistics 22.

We carried out six-fold cross-validation for each model with calculating the mean, standard deviation, and P-value of each indicator. As shown in **Table 1**, the better performance of the TabNet model on stability and robustness was observed compared with other models. As shown in **Table 2**, the importance of “Treatment protocols” has always ranked ahead of other variables.

**Table 1:** Prediction performance of different algorithms with six-fold cross-validation.

Metrics Algorithms	Dose regimen*^a^*	Precision (mean ± std, P)	Recall (mean ± std, P)	f1_score (mean ± std, P)	Support	Accuracy (mean ± std, P)	AUC (mean ± std, P)
LR	0	0.47 ± 0.34 (0.317)	0.15 ± 0.11 (0.785)	0.23 ± 0.16 (0.668)	11	0.68 ± 0.06 (0.463)	0.59 ± 0.11 (0.489)
	1	0.65 ± 0.03 (0.482)	0.91 ± 0.05 (0.317)	0.78 ± 0.04 (0.409)	19		
SVM	0	0.92 ± 0.09 (0.317)	0.31 ± 0.10 (0.585)	0.42 ± 0.14 (0.48)	11	0.71 ± 0.07 (0.405)	0.30 ± 0.13 (0.408)
	1	0.71 ± 0.06 (0.429)	0.99 ± 0.02 (0.317)	0.81 ± 0.04 (0.377)	19		
RF	0	0.81 ± 0.15 (0.317)	0.42 ± 0.18 (0.525)	0.56 ± 0.17 (0.484)	11	0.75 ± 0.05 (0.424)	0.79 ± 0.03 (0.397)
	1	0.73 ± 0.05 (0.418)	0.94 ± 0.04 (0.317)	0.83 ± 0.03 (0.388)	19		
AdaBoost	0	0.81 ± 0.07 (0.431)	0.42 ± 0.10 (0.435)	0.58 ± 0.07 (0.305)	11	0.76 ± 0.04 (0.418)	0.77 ± 0.06 (0.363)
	1	0.74 ± 0.06 (0.394)	0.92 ± 0.09 (0.373)	0.82 ± 0.09 (0.381)	19		
XGBoost	0	0.80 ± 0.14 (0.317)	0.44 ± 0.07 (0.585)	0.58 ± 0.09 (0.48)	11	0.76 ± 0.05 (0.405)	0.76 ± 0.04 (0.406)
	1	0.74 ± 0.03 (0.429)	0.93 ± 0.04 (0.317)	0.83 ± 0.04 (0.377)	19		
GBDT	0	0.77 ± 0.17 (0.317)	0.59 ± 0.14 (0.467)	0.67 ± 0.14 (0.4)	11	0.79 ± 0.08 (0.368)	0.81 ± 0.11 (0.357)
	1	0.79 ± 0.06 (0.388)	0.88 ± 0.06 (0.317)	0.84 ± 0.06 (0.354)	19		
LightGBM	0	0.69 ± 0.16 (0.391)	0.44 ± 0.07 (0.585)	0.54 ± 0.11 (0.505)	11	0.72 ± 0.08 (0.424)	0.76 ± 0.06 (0.413)
	1	0.74 ± 0.09 (0.434)	0.87 ± 0.12 (0.343)	0.80 ± 0.07 (0.391)	19		
CatBoost	0	0.84 ± 0.11 (0.317)	0.53 ± 0.11 (0.467)	0.65 ± 0.09 (0.4)	11	0.79 ± 0.09 (0.368)	0.78 ± 0.04 (0.362)
	1	0.77 ± 0.04 (0.388)	0.93 ± 0.05 (0.317)	0.85 ± 0.04 (0.354)	19		
TabNet	0	0.87 ± 0.03 (0.317)	0.64 ± 0.01 (0.525)	0.73 ± 0.01 (0.461)	11	0.82 ± 0.05 (0.405)	0.83 ± 0.01 (0.397)
	1	0.81 ± 0.02 (0.413)	0.95 ± 0.03 (0.317)	0.87 ± 0.02 (0.38)	19		
ANN	0	0.42 ± 0.11 (0.549)	0.45 ± 0.15 (0.585)	0.44 ± 0.13 (0.568)	11	0.55 ± 0.06 (0.484)	0.58 ± 0.07 (0.482)
	1	0.67 ± 0.05 (0.453)	0.54 ± 0.06 (0.43)	0.62 ± 0.04 (0.442)	19		
Super TML	0	0.78 ± 0.11 (0.427)	0.34 ± 0.11 (0.415)	0.48 ± 0.09 (0.361)	11	0.71 ± 0.05 (0.385)	0.56 ± 0.07 (0.354)
	1	0.70 ± 0.04 (0.323)	0.93 ± 0.05 (0.367)	0.80 ± 0.04 (0.381)	19		
Wide&Deep	0	0.69 ± 0.09 (0.549)	0.43 ± 0.12 (0.585)	0.54 ± 0.11 (0.568)	11	0.72 ± 0.07 (0.328)	0.74 ± 0.12 (0.389)
	1	0.72 ± 0.03 (0.355)	0.87 ± 0.04 (0.243)	0.79 ± 0.04 (0.355)	19		

^a^Regimen of 1,000 mg lapatinib corresponds to “0,” and regimen of 1,250 mg lapatinib corresponds to “1.”

**Table 2:** Feature importance from TabNet with six-fold cross-validation.

Feature	Importance (mean ± std)	P value
Treatment protocols	0.47 ± 0.05	0.612
Weight	0.23 ± 0.07	0.345
Number of chemotherapy treatments	0.16 ± 0.05	0.472
Number of metastases	0.05 ± 0.09	0.247

Treatment protocols including protocol_1 (combination regimen of lapatinib + capecitabine), protocol_2 (combination regimen of paclitaxel + carboplatin + herceptin + lapatinib), protocol_3 (combination regimen of vinorelbine + lapatinib), and protocol_4 (other combination regimens).

## Results

### Patients and Treatments

A total of 149 breast cancer patients were enrolled from FUSCC in this study; the population characteristics are illustrated in **Table 3**. There were 55 (36.90%) patients administered an initial lapatinib regimen of 1,000 mg, and 94 (63.10%) patients administered an initial lapatinib regimen of 1,250 mg. The median age of the patients was 51 years (interquartile range [IQR] 42~58 years), and 51.78% of patients were aged over 51 years. Patients using anthracycline, taxane, platinum, fluorouracil, and trastuzumab occupied 67.79%, 89.93%, 42.28%, 50.34%, and 91.28%, respectively. Comorbidities including hypertension, diabetes, and heart disease occupied 12.08%, 4.03%, and 3.36%, respectively. A percentage of 59.73% of patients were postmenopausal; 51.68% of patients had ≥3 previous chemotherapy regiments. Patients having ER and PR occupied 55.03% and 68.46%, respectively. A percentage of 75.84% of patients were in stage IV, 19.46% in stage III, and 4.70% in stage II. The most common metastatic site was in the lung (42.28%), bone (31.54%), liver (26.85%), and brain (18.12%). Patients using protocol_1, protocol_2, protocol_3, and protocol_4 were 67.11%, 16.11%, 6.71%, and 10.07%, respectively. There were 46 (30.87%) patients showing both safety and effectiveness of lapatinib treatment.

**Table 3:** Description of demographic and clinical characteristics.

Categories	Variables	Cases (N = 149)	Missing rate
Lapatinib information	Initial dose regimen, n (%)*^a^*		0
	0	55 (36.90%)	
	1	94 (63.10%)	
Demographic information	Age, year, median (IQR)	51 (42.0–58.0)	0
	Height, cm, median (IQR)	160.2 (158.0–162.0)	0
	Weight, kg, median (IQR)	58.3 (53.0–64.0)	0
	Age ≥ 52 years, n (%)	77 (51.78%)	0
Drug combination	Prior use of anthracycline, n (%)	101 (67.79%)	0
	Prior use of taxane, n (%)	134 (89.93%)	0
	Prior use of platinum, n (%)	63 (42.28%)	0
	Prior use of fluorouracil, n (%)	75 (50.34%)	0
	Prior use of trastuzumab, n (%)	136 (91.28%)	0
Physiopathological condition	Hypertension, n (%)	18 (12.08%)	0
	Diabetes, n (%)	6 (4.03%)	0
	Heart disease, n (%)	5 (3.36%)	0
	Other underlying diseases, n (%)	14 (9.4%)	0
	Postmenopausal, n (%)	89 (59.73%)	2.7%
Treatment information	Number of chemotherapy treatments, n (%)		0
	<3	72 (48.32%)	
	≥3	77 (51.68%)	
	Ki-67, median (IQR)	38.1 (20.0–50.0)	8.7%
	Prior endocrine therapy, n (%)	54 (36.24%)	0
	ER, n (%)		0
	0	67 (44.97%)	
	1	82 (55.03%)	
	PR, n (%)		0
	0	47 (31.54%)	
	1	102 (68.46%)	
	Stage, n (%)		0
	2	7 (4.70%)	
	3	29 (19.46%)	
	4	113 (75.84%)	
	Operation, n (%)		0
	0	13 (8.72%)	
	1	131 (87.92%)	
	2	2 (1.34%)	
	ECOG, n (%)		2.0%
	1	145 (97.32%)	
	2	4 (2.68%)	
	Number of metastases, n (%)		0
	0	36 (24.16%)	
	1	60 (40.27%)	
	2	33 (22.15%)	
	3	14 (9.04%)	
	4	6 (4.03%)	
	Metastases, n (%)		0
	<2	96 (64.43%)	
	≥2	53 (35.57%)	
	Lung metastases, n (%)	63 (42.28%)	0
	Liver metastases, n (%)	40 (26.85%)	0
	Bone metastases, n (%)	47 (31.54%)	0
	Brain metastases, n (%)	27 (18.12%)	0
	Other metastases, n (%)	35 (23.49%)	0
	Treatment protocols, n (%)*^b^*		0
	Protocol_1	100 (67.11%)	
	Protocol_2	24 (16.11%)	
	Protocol_3	10 (6.71%)	
	Protocol_4	15 (10.07%)	
Safety and effectiveness	Safety and effectiveness, n (%)*^c^*		0
	1	46 (30.87%)	
	0	103 (69.13%)	

^a^Regimen of 1250 mg lapatinib corresponds to “1,” and regimen of 1,000 mg lapatinib corresponds to “0.”

^b^Protocol_1 indicates combination regimen of lapatinib + capecitabine, protocol_2 indicates combination regimen of paclitaxel + carboplatin + herceptin + lapatinib, protocol_3 indicates combination regimen of vinorelbine + lapatinib, and protocol_4 indicates other combination regimens.

^c^Patient showing both safety and effectiveness corresponds to “1,” and other situations (either showing safety or effectiveness; not showing safety nor effectiveness) corresponds to “0.”

IQR, interquartile range; ER, estrogen receptors; PR, progesterone receptors; ECOG, Eastern Cooperative Oncology Group.

### Variable Selection

**Figure 2:**
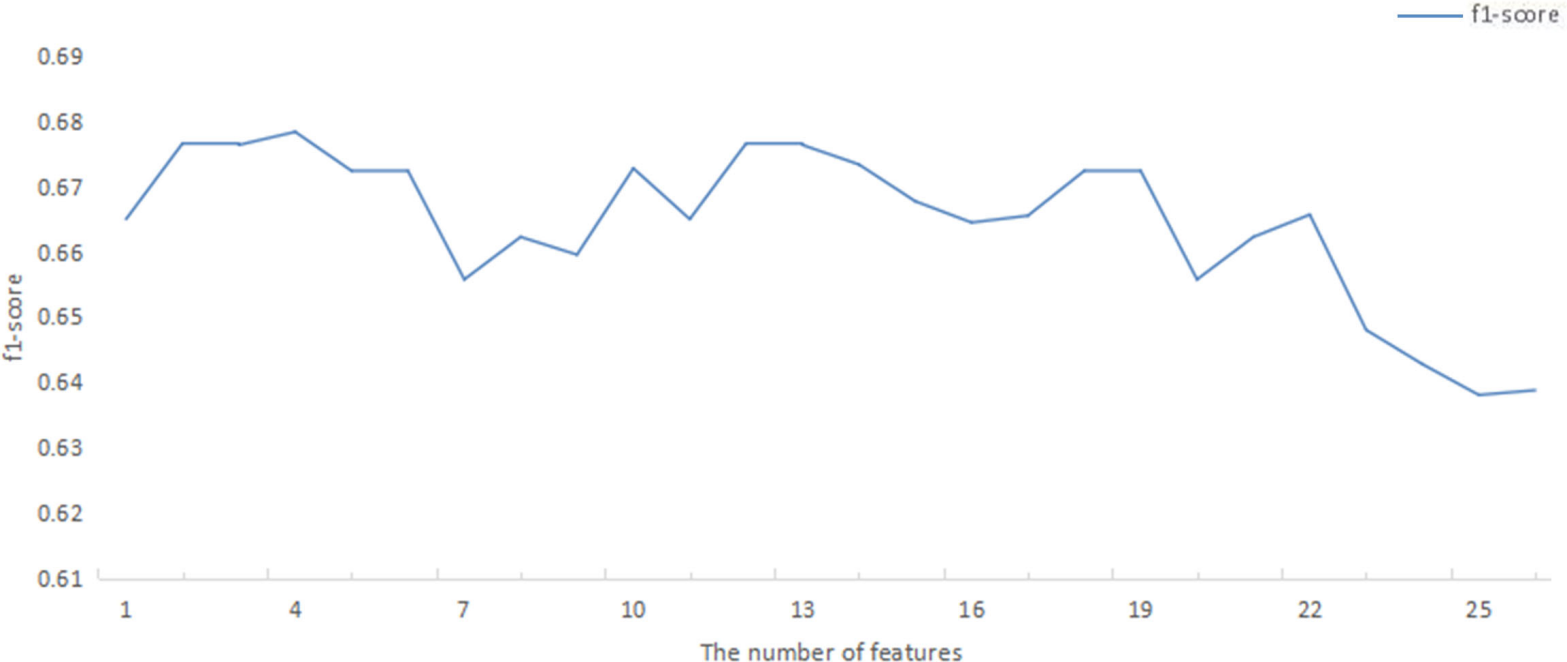
F1_score of RF model corresponding to the number of ranked variables. RF, random forest.

After deleting variables with extremely imbalanced positive and negative sample sizes (such as diabetes, heart disease, operation, and ECOG), features were selected based on 26 variables through the SFS method. RF models were established using the selected 1 to 26 variables, and the f1_score of each model was obtained (**Figure 2**). With increasing number of included variables, f1_score rises first and then reaches its maximum value at four variables (f1_score = 0.68). As we pursued a concise and accurate model with minimal variables but high predictive performance, the first four important variables were selected to establish the prediction model, including weight, number of chemotherapy treatments, number of metastases, and treatment protocols.

### Model Establishment

In **Table 1**, we presented the predictive performance of 12 models. TabNet had precision = 0.87, recall = 0.64, and f1_score = 0.73 for the 1,000-mg regimen prediction, and precision = 0.81, recall = 0.95, and f1_score = 0.87 for 1,250-mg regimen prediction, which indicate a comprehensive good predictive ability. In addition, accuracy = 0.82 and AUC = 0.83 for the whole TabNet model, which were higher than those for other algorithms. This shows that TabNet has competitiveness to predict the initial dose regimen of lapatinib accurately and establish a robust prediction model. On this basis, the importance scores of four selected variables were calculated and ranked by TabNet (**Table 2**). It can be seen that the most important variables were treatment protocols, weight, number of chemotherapy treatments, and number of metastases, with the importance scores of 0.47, 0.23, 0.16, and 0.05, in descending order. The P-values of treatment protocols, weight, and number of chemotherapy treatments were 0.612, 0.345, 0.472, and 0.247, respectively.

**Figure 3:**
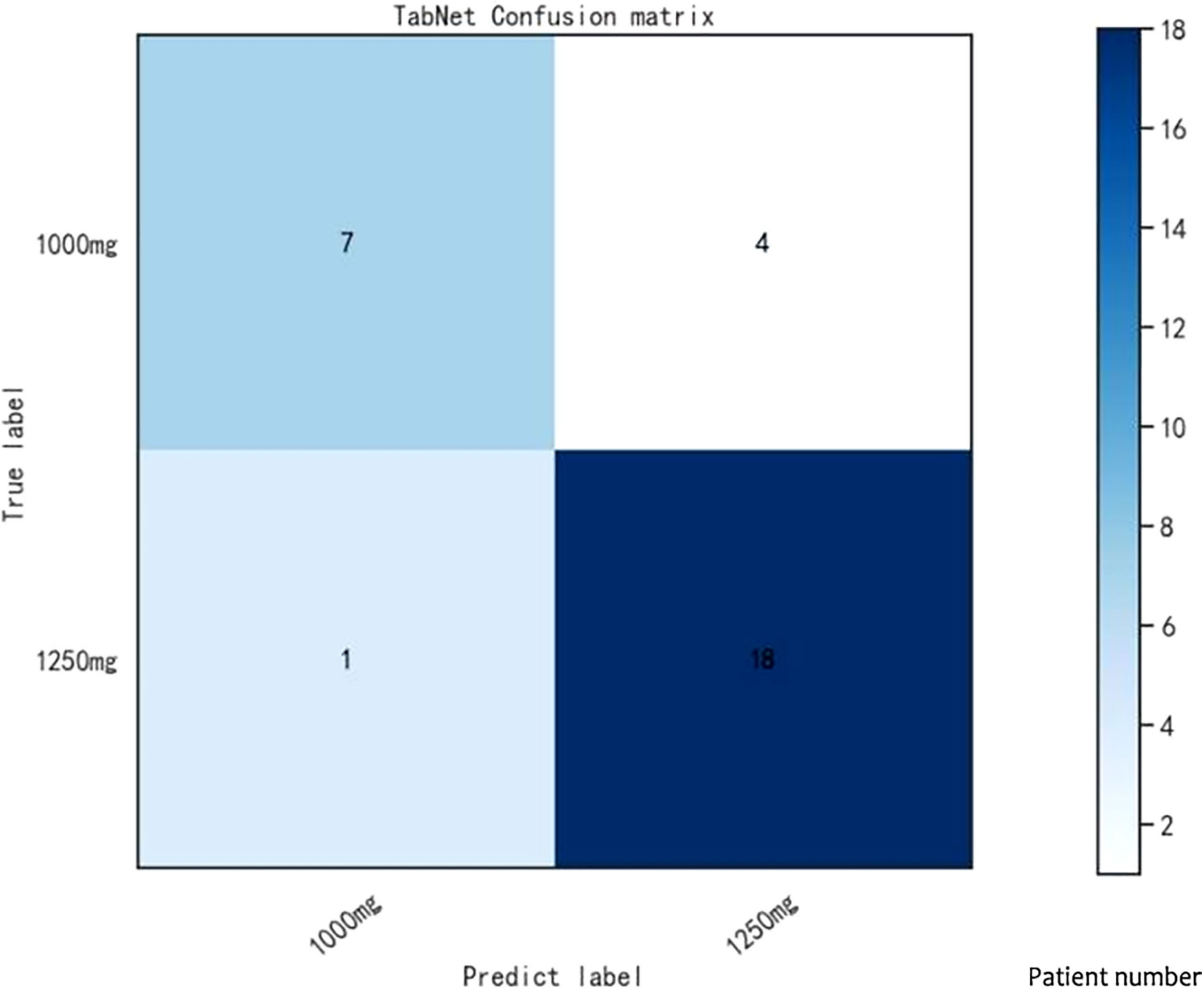
Confusion matrix in TabNet model.

The test cohort consisted of 30 patients, among which 19 patients took lapatinib 1,250 mg and 11 patients took lapatinib 1,000 mg. The dose of lapatinib was recommended for patients by establishing a confusion matrix based on the TabNet prediction model (**Figure 3**). The model recommended a dose regimen of 1,250 mg lapatinib accurately for 18 patients, and four patients were recommended the wrong dose, with a precision of 82% and a recall rate of 95%; the model recommended a dose regimen of 1,000 mg lapatinib accurately for seven patients, and one patient was recommended the wrong dose, with a precision of 88% and a recall rate of 64%.

## Discussion

Lapatinib was approved in China to treat patients with HER2(+) metastatic breast cancer in combination with capecitabine based on a single-arm, open-label study (EGF10949), which evaluates the drug efficacy and safety (1, 27). Our study focused on the establishment of a prediction model for lapatinib dose, mainly evaluating two dose regimens (1,000 and 1,250 mg). We used TabNet, a leading-edge deep learning technique, to construct the prediction model with good performance (accuracy = 0.82 and AUC = 0.83). Afterward, important variables that strongly correlated with lapatinib dose were ranked *via* an importance score, including treatment protocols, weight, number of chemotherapy treatments, and number of metastases. Lastly, the confusion matrix was used to validate the model; it can be seen that the dose regimen of 1,000 mg lapatinib had a precision of 88% and a recall rate of 64%, and the dose regimen of 1,250 mg lapatinib had a precision of 82% and a recall rate of 95%.

As a novel deep learning technique, TabNet uses a sequential attention mechanism to choose a subset of meaningful features to process at each decision step, enabling interpretability and more efficient learning as the learning capacity used for the most salient features (21). Additionally, based on retaining the end-to-end and representation learning characteristics of deep neural networks, TabNet also has the advantages of tree model interpretability and sparse feature selection (28). Other studies based on real-world evidence show that TabNet outperforms ensemble tree-based algorithms, since it can process a highly nonlinear relationship with its depth, without overfitting due to instance-wise feature selection (21). In this study, we applied TabNet, super TML, Wide&Deep, and ANN, which are algorithms with good predictive ability in various network algorithm types. After comparing the different network models, the TabNet model shows the best prediction performance (**Table S1**). Later, we will increase multicenter data. With larger data volume, we will build different network models and explore the optimum one to predict the dose of lapatinib to assist clinical medication.

As one of the most important variables influencing lapatinib dose, protocols of combination therapy accounted for a different proportion in this study as follows: protocol_1, protocol_2, protocol_3, and protocol_4 accounted for 67.11%, 16.11%, 6.71%, and 10.07%, respectively. The combination of lapatinib + capecitabine (protocol_1) was one of the common recommended regimens for patients with HER2(+) breast cancer with prior treatment of taxanes, anthracyclines, and/or trastuzumab (29, 30). Clinicians generally used lapatinib at a dose of 1,250 mg daily continuously plus capecitabine at a dose of 2,000 mg daily, and the combination can achieve superior treatment efficacy than capecitabine monotherapy (29, 31). In addition, a study on Japanese breast cancer patients found a drug–drug interaction and pharmacokinetics interaction between lapatinib and paclitaxel, as the AUC and Cmax of these patients given the combination therapy were affected (32). In Rezai et al.’s study, a pharmacokinetic interaction was found between lapatinib and vinorelbine, and the use of lapatinib can remarkably decrease the vinorelbine clearance (33). It has been proven that therapy of lapatinib combined with other drugs can commonly improve the time to progression and/or achieve longer survival time in these patients (29, 34, 35). Normally, previous studies focused on the efficacy and safety of combination therapy; few investigated the effect on dose regimen. Further research is needed to confirm the relationships between combination therapy and drug doses.

For the physiological features, previous studies found that patients with lower weight were more likely to have higher lapatinib plasma levels (P = 0.055) (36). The researchers believed that patients without fasting and lapatinib dose were not adjusted to low body weight during drug intake, which would lead to increased lapatinib body levels (36). The number of chemotherapy treatments was identified as an important influencing variable for lapatinib dose, and a previous study showed that three or more prior treatments strongly correlated with worse survival (1). Additionally, some studies found that organ metastases (such as liver and brain metastases) show a strong relationship with morbidity, mortality, and survival rate in breast cancer patients, which may affect the lapatinib dose regimen (37–39). In this study, the number of metastases has a remarkable effect on lapatinib dose whose correlation was previously investigated by few studies, which warranted further research.

According to the confusion matrix results, the classifier correctly identified 82% of patients using the 1,250-mg lapatinib regimen and 88% of patients using the 1,000-mg lapatinib regimen, indicating a remarkable prediction performance. Nevertheless, the sample size in the training and test cohorts was small. Large samples are required to verify this result.

## Conclusion

Our study endeavored to build a dose prediction model *via* machine learning and deep learning methods, which could mine deep data based on real-world evidence. Through a comparison of different algorithms, TabNet was selected to establish the model based on the strength of its predictive ability. To our knowledge, few studies focused on lapatinib dose prediction previously, and this study provides a new perspective and guidance for lapatinib dose administration with a more concise and accurate model. Compared with conventional models, machine learning and deep learning models mine and use unexploited variables to cover the shortage of clinical experience from the real world. One limitation in this study was the limited sample size that affected the model to further optimize the performance. In future, more real-world evidence should be added in the model to optimize its performance, and larger prospective clinical studies will be needed to investigate the further interactions between different variables and lapatinib dose.

## Data Availability Statement

The raw data supporting the conclusions of this article will be made available by the authors, without undue reservation.

## Ethics Statement

The studies involving human participants were reviewed and approved by the Ethics Committee at Fudan University Shanghai Cancer Center (FUSCC) (No. 2016-106-1159-K1). The patients/participants provided their written informed consent to participate in this study.

## Author Contributions

ZY and XY led the research and provided medical support. FK analyzed and interpreted the data. HYL, JZ, and ZW analyzed the data and provided technical support. HL and XH wrote the manuscript. HW and FG provided methodological guidance. QZ provided technical and medical guidance. All authors contributed to the article and approved the submitted version.

## Funding

FG was supported by the National Key Research and Development Program (2020YFC2005502, 2020YFC2005503). QZ was supported by the Clinical Research Plan of SHDC [SHDC2020CR3085B].

## Conflict of Interest

Author JZ and FG are employed by Beijing Medicinovo Technology Co. Ltd., China. Author XH is employed by Dalian Medicinovo Technology Co., Ltd., China.

The remaining authors declare that the research was conducted in the absence of any commercial or financial relationships that could be construed as a potential conflict of interest.

## Publisher’s Note

All claims expressed in this article are solely those of the authors and do not necessarily represent those of their affiliated organizations, or those of the publisher, the editors and the reviewers. Any product that may be evaluated in this article, or claim that may be made by its manufacturer, is not guaranteed or endorsed by the publisher.
