# Supplementary material for: Predicting Lapatinib Dose Regimen Using Machine Learning and Deep Learning Techniques Based on a Real-World Study
Source: Front Oncol. 2022 Jun 3;12:893966. doi: 10.3389/fonc.2022.893966 (PMC9203846; doi:10.3389/fonc.2022.893966)
Supplement: Supplementary file 1 [file DataSheet_1.docx]

Supplementary Figures

Figure S1. The encoder of the TabNet architecture

Figure S2. The Decoder of the TabNet architecture

Figure S3. The topological structure of the Feature Transformer layer

Figure S4. The topological structure of the Attentive Transformer layer
